# Modifiable Risk Factors associated with Post-Operative Bleeding and transfusion requirements in Cardiac Surgery

**DOI:** 10.12669/pjms.38.4.5685

**Published:** 2022

**Authors:** Bahauddin Khan, Mujahid Ul Islam, Imtiaz Ahmad, Mujeeb Ur Rehman

**Affiliations:** 1 Dr. Bahauddin Khan, FCPS. Assistant Professor, Department of Cardiothoracic Surgery, Rehman Medical Institute, Peshawar, Pakistan; 2 Dr. Mujahid Ul Islam, FCPS. Associate Professor, Department of Anesthesia, Rehman Medical Institute, Peshawar, Pakistan; 3 Dr. Imtiaz Ahmad, FCPS. Associate Professor, Department of Anesthesia, Rehman Medical Institute, Peshawar, Pakistan; 4 Dr. Mujeeb Ur Rehman, MS. Senior Registrar, Department of Cardiovascular Surgery, Lady Reading Hospital, Peshawar, Pakistan

**Keywords:** Bleeding, Cardiopulmonary bypass, Open heart surgery, Hemoglobin, Platelets

## Abstract

**Objectives:**

In this study we determine the modifiable factors related to bleeding and transfusion in post-cardiac surgery patients who underwent open heart surgery.

**Methods:**

This is a retrospective study that include two hundred patients who had undergone open heart surgery (OHS) at Northwest General Hospital and Research Center from December 2018 to July 2021. Platelet count and hemoglobin level were measured in the pre-operative period.

**Results:**

This study included both male and female patients. Postoperative platelets were counted as follow: 50-100 x10^9^ L in 3.0% cases, 101-150 x10^9^ L seen in 27.5% cases, and >150 x 10^9^ L in 69.5% cases which required transfusion. We have also reported the increased requirement of transfusion of blood and blood products in patients with pre-operative hemoglobin (Hb) < 10 g/dl.

**Conclusion:**

Correction of pre-op Hb, post-op platelet count and total bypass time are the significant and preventable parameters in patients undergoing cardiac surgery if proper pre-op assessment of the patient is performed.

## INTRODUCTION

Bleeding and transfusion of blood and blood products is a serious concern in patients undergoing cardiac surgery.^1^ Some of the significant factors including deficiency of coagulation factor, an improper heparin reversal, raised fibrinolytic state, platelet deficiency and technical causes. The surgical technical causes have been reported the leading cause of coagulopathic condition.^1^-^3^ The definite cause of bleeding is not easy to define. This is started with replacement of volume and transfusion of blood, FFP and platelet transfusion, infusion of tranexamic acid. In certain cases, correction of acidosis, regulation of blood glucose level, reversal of hypothermia, mechanical and chemical inotropic support standardization, correction and monitoring of cardiac rhythm are important to prevent bleeding.^4^,^5^ Use of Thrombo Elastography (TEG) is important to know the underlying pathology. The ultimate option to identify and treat the factor of bleeding in 2 to 11% of cases is chest exploration.^6^ Most common causes of Excessive postoperative bleeding (75%) of cases are from surgical origins.^6^,^7^

The surgical factor of bleeding requiring re-exploration reported from 35-100%.^8^ It is usually at the sites of anastomosis, conduits side branches, soft tissues under the sternum, suture sites of the sternum, periosteum and bone marrow.^2^ Anti-platelet medications are also leading to platelet defects in acute coronary disease patients resulting in platelet dysfunction in Preoperative condition.^8^ Clopidogrel and Aspirin are the commonly used drugs in cardiac surgery patients.^5^ Increase re-exploration rate has been reported in Clopidogrel taking patients.^9^ Preoperative Platelet count (< 100 x 10^9^ ) L is a dreadful cause of postoperative bleeding and blood transfusion requirements.^10^ Cardiopulmonary bypass machine has been shown the significant cause of intraoperative coagulopathy.^8^ It is reported that Prolonged cross-clamp time is the significant factor of profuse post-op bleeding. Body temperature of 33 Celsius or less affects the adhesion and Platelet aggregation. The “heparin rebound” phenomenon has reported a significant factor of immediate post-op bleeding.^11^ Blood transfusion is a life-saving intervention in active postoperative bleeding. Blood transfusion is having adverse reactions, such as volume overload, hypothermia, potassium, calcium, magnesium-related disorders, acute lung injury, renal damage, infection transmission.^12^ The intensity and frequency of these complications are leading to the number of units transfused. Therefore, factors promoting bleeders should be controlled in the preoperative, intraoperative, and post-operative period.^13^

Preoperative clopidogrel and warfarin cessation, treating infections, avoiding surgery immediately after thrombolysed patients, correcting anemia, avoiding surgery in menstruating females, optimizing liver function, diagnosing and preparation for rare coagulation anomalies, management etc., are some common causes which should be corrected, if possible, prior to surgery.^13^,^14^

Postoperative blood transfusion, FFPs, platelets concentrate, tranexamic acid and High PEEP for the bleeders to get tamponade, temperature regulation, correcting the acidosis are the concern for the excessive bleeding.^14^-^17^ If there is hemodynamics instability then early and immediate chest re-exploration should be considered to remove the clot in the chest. Early reopening has been considered to avoid extra blood transfusions and the patient’s physiological reserve.^18^

The clinical significance of this study is; Pre-op Hb, platelets count and bypass pass time have been shown that greatly affect bleeding and transfusion requirement in post-op cardiac patients.

## METHODS

The retrospective study was conducted in the cardiac surgery unit at North West General Hospital and Research Center, Peshawar from December 2018 to July 2021. A total of two hundred patients underwent open heart surgery during this period. After acquiring ethical approval from the research and ethical committee (Ref# NwGH/Res/Ethical Approval/1441, of the institute. data was retrieved from Hospital Management Information System (HMIS). Pre-op Hb, post-op platelet count, and total bypass time are analyzed in this study.

Patients having both genders, of all ages, undergoing coronary artery bypass grafting (CABG), valve surgery (MVR, AVR, DVR), Congenital corrective procedures (ASD, VSD, TC) and off-pump bypass grafting were included in the study. We also included the patients presenting for elective cardiac surgery, with or without CPB respectively. On the other hand, patients with Endovascular procedures (trans-aortic valve replacement (TAVR) and all percutaneous techniques), non-cardiac thoracic operations and surgeries on the descending aorta were not included in this study.

SPSS (statistical package for social sciences) version 22 was used for data analysis. Descriptive statistics (mean, frequencies and percentage) were applied to analyze data of the study subjects. Frequencies and percentage of the study variables were calculated and data represented graphically (bar graph, pie chart etc.) as well as in the description.

## RESULTS

**Fig.1:**
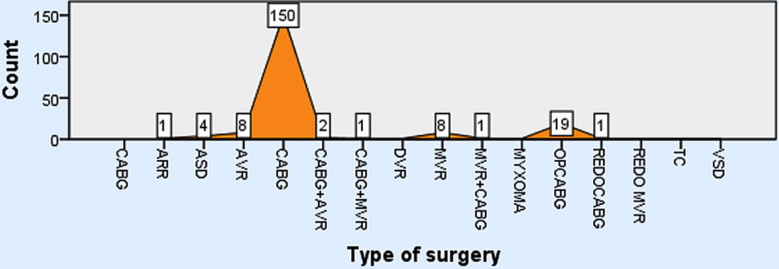
Procedures Breakup. ***Abbreviation:*** ASD: Atrial Septal Defect, VSD: Ventricular Septal Defect, AVR: Aortic Valve, Replacement, DVR: Double Valve Replacement, OPCABG: Off-Pump Coronary Artery Bypass Grafting, MVR: Mitral Valve Replacement, TC: Total Correction, REDO: Re-Opening cases

Age distribution breakup of 200 patients was as follows, 4 (2.0%) patients were between 0-15 years, between 16-30 years fell 6 (3.0%) patients, 31-45 year were 34 (17.0%) patients, 46-60 years were 93 (46.5%) patients, and from age 61-75 years were 63 (31.5%) patients. Gender distribution were 30.50% (Male) and 69.50% (Female). Most common Procedure was CABG as shown in Fig.1. Postoperative platelets count 50-100 x109L in 3.0% cases, 101-150 x109L seen in 27.5% cases, >150 x 109L count seen in 69.5% cases.

The Pre-operative Hb level has been shown in Table-I and was commonly more than 14 g/dl in (61%) patients and was less than 11g/dl in less number of patients such as 10.0-11.0 g/dl in 3.0% and <10 g/dl in 1.5% patients. Low pre-op Hb resulting in increased post-op transfusion requirements as shown in Table II. 61% patients with pre-op Hb > 14 did not require any post-op transfusion. There were maximum number of patients (17.0%) with Hb 13.1 _ 14.0, who received one transfusion. Maximum number of transfusions have been given to patients with Hb < 10. This table is based on the number of transfusions on the basis of Hb. Table-II

**Table-I:** Association of Preoperative Hemoglobin Level with post-op transfusion.

Hemoglobin range(g/dl)	Frequency	Percentage	Number of transfusions
< 10	3	1.5%	5
10.0 _ 11.0	6	3.0%	4
11.1 _ 12.0	14	7.0%	3
12.1 _ 13.0	21	10.5%	2
13.1 _ 14.0	34	17.0%	1
> 14	122	61.0%	0

Total	200	100.0%	

PRE-OP Hb; Pre-operative hemoglobin, POST-OP; post-operative.

**Table-II:** Patient’s characteristics.

Number of Transfusion	Frequency	Cumulative Percent
0	89	44.5%
1	52	26%
2	35	17.5%
3	15	7.5%
4	6	3%
5	3	1.5%

Total	200	100%

As shown in Table-I, 89 (44.5%) had no blood transfusion, 52 (26.0%) had 1 unit, 35 (17.5%) had 2 units, 15 (7.5%) had 3 units, 6 (3.0%) had 4 units, and 3 (1.5%) had 5 units.

As can be seen in Table-III, total blood volume lost is proportional to the bypass time which perfectly corresponds to published literature.. Bypass time is a significant factor of bleeding in cardiac surgery patients as it greatly affects the platelet count. Table-IV There were 85.0% patients with PLT count of >150x 10^9^L in OPCAB patients and PLT count (>150 x 109)L was shown in 60.0% of patients with bypass time of >120 minutes. Moreover, the PLT count was (>150 x 109) L in 76.5% patients with bypass time <60 minutes.

**Table-III:** Association of Total Bypass time with Post-operative Bleeding.

Post-operative bleeding	Total Bypass time				Total

	<60	61-120	>120	OPCABG	
0-250	5.9%	1.4%			1.5%
251-500	11.8%	17.6%	6.7%	25.0%	17.0%
501-750	41.2%	50.7%	80.0%	55.0%	52.5%
>750	41.2%	30.4%	13.3%	20.0%	29.0%

Total	100.0%	100.0%	100.0%	100.0%	100.0%

**Table-IV:** Association of Total Bypass time with Post-operative platelets count.

Post-operative platelets count		Total Bypass time				Total

		<60	61-120	>120	OPCAB	
	50 -100		4.1%			3.0%
	101-150	23.5%	28.4%	40.0%	15.0%	27.5%
	>150	76.5%	67.6%	60.0%	85.0%	69.5%

Total		100.0%	100.0%	100.0%	100.0%	100.0%

OPCAB; Off-pump Coronary Artery Bypass.

**Fig.2:**
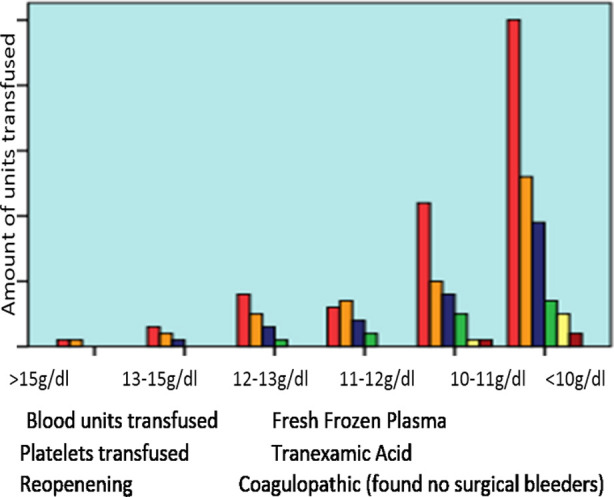
Association of Pre-Operative Hemoglobin Level with chest re-opening, post-op blood and blood products requirements’

Our study has further illustrated that hemoglobin level alone has a major effect on post-operative transfusion requirement, fresh frozen plasma requirement, platelets transfusion requirement, chest reopening requirement, and coagulopathy (Fig.2).

With preoperative hemoglobin above 15g/dl, only one patient required blood and fresh frozen plasma transfusion, as pre-operative hemoglobin level decreases the amount of blood and blood products required also increased. Below 10g/dl pre-operative levels the requirement of blood and blood products are common as opposed to more than 15g/dl.

## DISCUSSION

Bleeding Academic Research Consortium (BARC reported that 5 or more units of packed red blood cells (PRBCs) transfusions in 48 Hours and greater than 2 liters of chest tube output in 24 Hours are said to be significant in cardiac surgery patients.^5^ Patients that bleed actively can be taken to these summits rarely and early steps for optimization are usually necessary including the use of blood products, blood, medical optimization and reoperation. This is only possible with proper preoperative assessment of the patient.^6^ Correction of acidosis and hypothermia are the entity leading to decrease bleeding. The small bleeders can be tamponade by keeping the PEEP of around 10cm. These parameters can be achieved in order to assess the cause and status of profuse bleeding such as complete blood count, Thromboelastography, Prothrombin time, Activated thromboplastin time and fibrinogen levels.^6^,^11^ To improve the coagulation, Platelets transfusion, Fresh Frozen Plasma, and occasionally Desmopressin are the important parameters. Additional Protamine can be used to treat the Heparin rebounding phenomenon. Coagulopathy can be treated with a novel agent such as Recombinant factor VII. Aminocaproic acid and Tranexamic acid have frequent use too.^19^ The Commonly used and avoiding Preoperative associated factors leading to postoperative bleeding are Clopidogrel which should be stopped five days before the surgery, Ticagrelor which should be stopped three days before the operation, Heparin for acute coronary syndromes, low molecular weight heparin and patients on Warfarin or novel oral anticoagulants as these factors are not analyzed in our study. Common causes of excessive bleeding are extra cardiac bleeders, LIMA bed, and surgical bleeders from either distal or proximal anastomosis sites. That is why; it is quite complicated to ensure proper hemostasis.^11^

Different Intraoperative common methods to avoid bleeding include clip application, stitching, and ligation for significant identified points. It is reported that the success rate of intraoperative Fibrin sealant or topical hemostatic agents is 92.6%.^16^ It has been suggested that delayed sternal wound closure will get rid of later clot removal and tamponade.^18^

Multiple Causes of excessive bleeding have been reported recently in cardiac surgery patients such as 74% cases are due to technical or mechanical reasons, coagulopathy (13%), combination (10%) and others 3%.^6^-^10^ Technical factors are the most common reasons for reoperations for bleeding.^20^

The function of preoperative hemoglobin is well identified in recognizing the significance of factors related to adverse outcomes.^21^ Assessing the quality of the preoperative platelets in open-heart surgery patients would decrease the frequency of hemorrhage and blood transfusion.^22^ A recently reported meta-analysis, that included >3,000 patients reported that platelet dysfunction in patients undergoing cardiac surgery can be determine by Point of Care (POC) platelet function tests perioperatively.^22^,^23^ Platelet function and coagulation system are strongly affected by Cardiopulmonary bypass (CPB) via different mechanisms, like hemodilution leading to decrease platelet count, artificial surfaces resulting in contact activation, bypass-induced platelet activation, massive doses of heparin, reversal of heparin with protamine, hypothermia and reduced hemoglobin.^24^,^25^

Recently reported studies stated that monitoring of platelet function are feasible and beneficial during and after CPB.^26^-^28^ ADP activation test might be useful to determine the CPB effect on platelet function.^29^ Assessment of preoperative platelet function would result in decrease frequency of postoperative bleeding and blood transfusion as compared to those of the postoperative evaluation.^25^ It has been reported in a local study that bypass time doesn’t affect the post-op platelets.^30^ But our study reported that total bypass time has a significant effect on postoperative platelet count as shown in Table-IV as the amount of platelet decreased by increasing the bypass time and vice versa.

Platelet dysfunction is a significant cause of postoperative bleeding in cardiac surgery patients. The association between platelet function and severe bleeding was defined by Operating Room (PLATFORM) study after CPB, giving the platelet function adequate predictive values for massive bleeding. A cohort trial on 490 adult cardiac surgery patients with CPB reported that TRAPtest and ADPtest post-CPB had significant massive bleeding (P = 0.001).^31^ we did not use this test and will consider it for future studies.

Platelet dysfunction in cardiac surgery resulting in bleeding due to contact with the surfaces of non-biological cardiopulmonary bypass (CPB) machine.^26^ However, platelets permit the shear the pumping of blood poorly, getting partially activated and shed the contents of their granules.^32^ The platelet count decreases during the course of the surgical procedure due to their consumption in the circuit resulting in impaired function of the remaining platelets. Hence, platelet function defect and thrombocytopenia are commonly treated with transfusions of platelet in patients undergoing CPB.^33^

Recently reported study on 177 patients observed thrombocytopenia in 167(94.4%) patients from the baseline value. There was maximum drop in platelet count during 2nd and 3rd postoperative days. Hence, severe thrombocytopenia (<50,000 x 10^9^/l) was observed in 9(5.3%) patients.^34^ Allogeneic blood transfusion has been reported as an associated factor for hospital-acquired infection, suppression of immune, overload of circulatory system and mortality. it is also stated that tissues ischemia may occur due to restricting RBC transfusion, ultimately affecting the prognosis.

The trigger of the liberal and restrictive RBC transfusion in the high-risk cardiac surgery patients (n=5,243) has been only discovered by the Transfusion Requirements in Cardiac Surgery TRICS-III trial. Cardiac surgery patients were randomized in the preoperative state to a restrictive [hemoglobin (Hgb) <7.5 g/dL] or liberal transfusion trigger (Hgb <9.5 g/dL in the operating theater and ICT, and Hgb <8.5. The primary leading cause of myocardial infarction, stroke, death and new-onset of renal failure requiring dialysis in the restrictive group had been found non-dominant to the liberal group (11.4% restrictive vs. 12.5% liberal P<0.001).^35^

It is true that blood transfusion does save many lives and it has always been a concern in cardiac surgery where blood loss is often seen. Blood is a scarce factor, and its complications must compare against the benefits. However, there is a need for the protocols of transfusion as blood transfusion increase mortality in critically ill patients.^36^ It has also been shown that blood transfusion shift the prognosis and outcome in anemic patients.^37^ many studies have calculated the risk of morbidity and mortality.^38^ According to recent studies, more conservative blood transfusion protocols are being used in various centers.^39^ Postoperative Infection has been reported the associated controversial risk with blood transfusion.^40^

It has been reported that preoperative anemia, morbidity, mortality, and higher hospital costs are associated with increased blood transfusions.^41^ Higher rates of stroke, acute renal injury, and total number of adverse postoperative outcomes have also been reported to be associated with preoperative anemia in cardiac surgery patients.^42^

Our study reported that Pre-op Hb <10g/dl required increase number of blood and blood products transfusion as compared to pre-op Hb 13-15g/dl or >15g/dl. Our study has also showed that the requirement of postoperative blood transfusion, platelets transfusion, fresh frozen plasma, coagulopathy and chest reopening are greatly affected by preoperative hemoglobin level alone. Post-op infection and other post-op transfusion-related complications data have not been collected. It is worth noting at this point that a multicenter prospective study would be required to further evaluate our findings.

### Limitation of the study:

This study has small sample size and single centered. Our study does not include the association of post-op bleeding with age, gender, pre-op platelets and weight in cardiac surgery patients. We have included all the open heart surgery patients in our study. Multi-center studies will be needed to know the post-op bleeding in individual open heart surgery patients. We did not note the details of pre-op anti platelet and anti-coagulant medications as well.

## CONCLUSIONS

The preoperative evaluation and recognition of Hb, post-op platelet quantity and function, and decreasing the total bypass time are the significant and preventable parameters related to post-operative bleeding and transfusion requirement in cardiac surgery patients. It will also decrease the ICU and hospital stay as well.

### Authors’ Contribution:

**MUR & BK:** Designed and conceived the study, data analysis and editing.

**MUI:** Helped in data collection and manuscript writing.

**IA:** Helped in data collection, statistical analysis.

**MUR:** Analysis of data and final approval.

**MUR:** Responsible and accountable for the accuracy and integrity of the work.
